# Phos-tag-based approach to study protein phosphorylation in the thylakoid membrane

**DOI:** 10.1007/s11120-020-00803-1

**Published:** 2020-12-02

**Authors:** Keiji Nishioka, Yusuke Kato, Shin-ichiro Ozawa, Yuichiro Takahashi, Wataru Sakamoto

**Affiliations:** 1 Institute of Plant Science and Resources (IPSR), Okayama University, 2-20-1 Chuo, Kurashiki, Okayama 710-0046 Japan; 2 Research Institute for Interdisciplinary Science, Okayama University, 3-1-1 Tsushima-naka, Kita-ku, Okayama City, Okayama 700-8530 Japan; 3 Present Address: Faculty of Agriculture, Setsunan University, 45-1 Nagaotoge-cho, Hirakata, Osaka 573-0101 Japan

**Keywords:** Chloroplast, Phos-tag, Protein phosphorylation, Thylakoid membrane, STN7, STN8

## Abstract

Protein phosphorylation is a fundamental post-translational modification in all organisms. In photoautotrophic organisms, protein phosphorylation is essential for the fine-tuning of photosynthesis. The reversible phosphorylation of the photosystem II (PSII) core and the light-harvesting complex of PSII (LHCII) contribute to the regulation of photosynthetic activities. Besides the phosphorylation of these major proteins, recent phosphoproteomic analyses have revealed that several proteins are phosphorylated in the thylakoid membrane. In this study, we utilized the Phos-tag technology for a comprehensive assessment of protein phosphorylation in the thylakoid membrane of *Arabidopsis*. Phos-tag SDS-PAGE enables the mobility shift of phosphorylated proteins compared with their non-phosphorylated isoform, thus differentiating phosphorylated proteins from their non-phosphorylated isoforms. We extrapolated this technique to two-dimensional (2D) SDS-PAGE for detecting protein phosphorylation in the thylakoid membrane. Thylakoid proteins were separated in the first dimension by conventional SDS-PAGE and in the second dimension by Phos-tag SDS-PAGE. In addition to the isolation of major phosphorylated photosynthesis-related proteins, 2D Phos-tag SDS-PAGE enabled the detection of several minor phosphorylated proteins in the thylakoid membrane. The analysis of the thylakoid kinase mutants demonstrated that light-dependent protein phosphorylation was mainly restricted to the phosphorylation of the PSII core and LHCII proteins. Furthermore, we assessed the phosphorylation states of the structural domains of the thylakoid membrane, grana core, grana margin, and stroma lamella. Overall, these results demonstrated that Phos-tag SDS-PAGE is a useful biochemical tool for studying in vivo protein phosphorylation in the thylakoid membrane protein.

**Supplementary Information:**

The online version of this article (10.1007/s11120-020-00803-1) contains supplementary material, which is available to authorized users.

## Introduction

Protein phosphorylation is the most common post-translational modification that regulates and/or fine tunes protein functions. Proteins are phosphorylated by protein kinases in a position-dependent manner, and they are reversibly dephosphorylated by protein phosphatases. In the chloroplast, numerous phosphorylation sites in photosynthesis-related proteins in the thylakoid membrane have been identified (Grieco et al. 2016). Recent studies have revealed that the reversible phosphorylation of the photosystem II (PSII) complex and light-harvesting complex of PSII (LHCII) is important to overcome light stress (Pesaresi et al. 2011; Tikkanen and Aro 2012; Allahverdiyeva et al. 2015). The phosphorylation of the LHCII trimeric complex, composed of Lhcb1, Lhcb2, and Lhcb3, drives state transition, which is an acclimation mechanism to balance excitation pressure between the PSII and photosystem I (PSI). Phosphorylated LHCII has been demonstrated to be relocated from the PSII to PSI under high-light conditions. Conversely, dephosphorylated LHCII is transported back to the PSII for balancing light absorption capacity between the PSII and PSI. In *Arabidopsis*, STN7 kinase is known to regulate the phosphorylation of LHCII (Bellafiore et al. 2005; Bonardi et al. 2005), whereas PP2C-type phosphatase (PPH1)/thylakoid-associated phosphatase of 38 kDa (TAP38) regulates the dephosphorylation of LHCII (Shapiguzov et al. 2010; Pribil et al. 2010). On the contrary, the phosphorylation of D1, one of the core subunits of the PSII complex (also called PsbA), appears to be involved in fine-tuning the photo-damaged D1 turnover in the PSII repair cycle (Baena-González and Aro 2002; Fristedt et al. 2009; Kato and Sakamoto 2009, 2018). STN8 kinase regulates the phosphorylation of D1, which is dephosphorylated by PSII core phosphatase (PBCP) (Bonardi et al. 2005; Samol et al. 2012). These phosphorylation states of protein complexes are expected to change the structural domains of the thylakoid membrane, grana core (appressed region), grana margin, end membrane, and stroma lamella (non-appressed region). For instance, phosphorylated LHCII migrates from the grana core, in which the PSII is mostly localized, to the grana margin and stroma lamella, in which the PSI is rich during state transition (Dumas et al. 2016). However, the phosphorylation state of most of the thylakoid membrane proteins, according to their spatial distribution within the thylakoid membrane, remains to be elucidated.

In this study, we attempted to exploit the Phos-tag technology, a relatively simple method to distinguish phosphorylated proteins from their non-phosphorylated isoforms, to characterize the phosphorylated proteins in the thylakoid membrane. Phos-tag, a dinuclear metal complex of the 1,3-bis[bis(pyridin-2-ylmethyl)amino]propan-2-olato dizinc (II) complex, is a phosphate-binding molecule, and it has been developed as a tool to visualize phosphoproteins (Kinoshita et al. 2004, 2005). The Phos-tag technology is utilized in various phophoprotein-trapping techniques, including SDS-PAGE. In Phos-tag SDS-PAGE, Phos-tag interferes with the migration of phosphorylated proteins in the gel, resulting in the separation of phosphorylated proteins as a retarded band from their non-phosphorylated forms, depending on the phosphorylation states. Indeed, this technology can separate phosphorylated proteins and non-phosphorylated proteins in the thylakoid membrane (Crepin and Caffarri 2015; Longoni et al. 2015). In this study, to the best of our knowledge, we first employed two-dimensional (2D) SDS-PAGE to separate the thylakoid membrane proteins (2D Phos-tag SDS-PAGE). Thylakoid proteins were separated in the first dimension by conventional SDS-PAGE and in the second dimension by Zn^2+^-Phos-tag SDS-PAGE; phosphorylated proteins were detected as spots that showed retarded migration in the second dimension. Subsequently, protein spots that resulted from 2D Phos-tag SDS-PAGE were identified by liquid chromatography-tandem mass spectrometry (LC–MS/MS). Furthermore, we extended the Phos-tag assay to examine the phosphorylation state of photosynthesis-related proteins in the structural domains of the thylakoid membrane, such as the grana stack, grana margin, and stroma lamella. The differences in the phosphorylation status of several photosynthesis-related proteins between the thylakoid subfractions, as determined by Phos-tag SDS-PAGE and immunoblotting, suggested the regulation of photosynthesis-related proteins by protein phosphorylation in the different structural domains.

## Materials and methods

### Plant materials and growth conditions

*Arabidopsis thaliana* ecotype Columbia (Col) was used as the wild type (WT). The *stn7* (SALK073254) and *stn8* (SALK064913) mutants in the genetic background of *Arabidopsis* ecotype Col-0 were also used in this study. The double mutant *stn7stn8* was generated by crossing these mutants. The plants were grown on plates or soil. For growth in plates, the seedlings were grown for 3 weeks on 0.7% (w/v) agar plates containing Murashige and Skoog (MS) medium supplemented with 1.5% (w/v) sucrose at 23 °C under white LED light (160 μmol photons m^−2^ s^−1^) with 10 h of daylight. For growth in soil, *Arabidopsis* seeds were directly sown in soil-filled polyethylene plant pots. For high-intensity light irradiation, the plants were exposed to white LED light (1200 μmol photons m^−2^ s^−1^) for 2 h.

### Isolation of the thylakoid membrane

The thylakoid membrane was purified from 3-week-old seedlings or leaves of 2-month-old mature plants. The seedlings on MS plates were collected and ground in 50 mL of ice-cold EDTA-free thylakoid isolation buffer (350 mM sucrose, 50 mM HEPES–NaOH (pH 7.0), 5 mM 1 M MgCl_2_, and 10 mM 5 M NaCl) with an Iwatani millser (Iwatani Corporation, Tokyo, Japan). Phosphatase inhibitors (10 mM NaF and 0.5 mM NaVO_3_) were added to the buffer before use. The lysate was filtered using Miracloth (EMD Millipore Corporation, Burlington, MA, USA) and then centrifuged at 2100 × *g* for 10 min at 4 °C. The pellet was resuspended in 50 mL of thylakoid isolation buffer and centrifuged at 300 × *g* for 1 min at 4 °C. The supernatant was transferred to a new conical centrifuge tube and centrifuged at 2100 × *g* for 10 min at 4 °C. The pellet was resuspended in 1 mL of 50 mM HEPES of pH 7.6. To measure chlorophyll concentration, 20 μL of the suspension was added to 1 mL of 80% (v/v) acetone and centrifuged at 15,000 × *g* for 1 min. The supernatant was measured using a spectrophotometer, Ultrospec 2100 pro (Amersham Biosciences, Little Chalfont, UK), at wavelengths of 646 and 663 nm (wavelengths of chlorophyll). Chl concentration was calculated as 0.905 × {(A_646_) − (A_750_)} + 0.374 × {(A_663_) − (A_750_)}, according to Porra et al. (1989).

### Two-dimensional Phos-tag SDS-PAGE

The phosphorylation of thylakoid membrane proteins was analyzed by two-dimensional (2D) Phos-tag SDS-PAGE. The proteins were separated by conventional SDS-PAGE in the first dimension and were separated by Zn^2+^-Phos-tag SDS-PAGE in the second dimension. In brief, conventional SDS-PAGE was performed using a slab size electrophoresis system (138 mm × 130 mm × 1 mm; ATTO Corporation, Tokyo, Japan) in the first and second dimensions. The Phos-tag gel consisted of a separating gel [10% (w/v) acrylamide, 350 mM Bis–Tris (pH 6.8), 25 μM Phos-tag acrylamide, 100 μM ZnCl_2_, 0.1% (v/v) N,N,N′,N′-tetramethylethylenediamine (TEMED), and 0.05% (w/v) ammonium persulfate (APS)] and a stacking gel [4.5% (w/v) acrylamide, 350 mM Bis–Tris (pH 6.8), 0.1% (v/v) TEMED, and 0.05% (w/v) APS]. The purified thylakoid proteins were solubilized in SDS-PAGE sample buffer at 37 °C for 30 min, and were then loaded, based on equal chlorophyll content (2.5 μg Chl.). Electrophoresis of the first dimension was performed at a constant current of 30 mA/gel. After electrophoresis, the gel was cut into slices and equilibrated in SDS-PAGE sample buffer at 37 °C for 30 min. The gel slices were placed on top of the second-dimension gel and embedded in 1% (w/v) agar with Phos-tag SDS-PAGE running buffer. The gel slices were also subjected to Coomassie brilliant blue R-250 (CBB) staining to show protein separation in the first dimension. Electrophoresis was performed at a constant current of ≤ 20 mA/gel with the running buffer [100 mM Tris, 100 mM MOPS, and 0.1% (w/v) SDS], supplemented with 5 mM sodium bisulfite. The gel was silver-stained using the Sil-Best Stain One kit (Nacalai Tesque, Kyoto, Japan) according to the manufacturer’s instructions. Protein Ladder One, Triple-color (Broad Range) for SDS-PAGE (Nacalai Tesque, Kyoto, Japan), was used as a molecular marker in both first and second dimensions.

### Pro-Q Diamond staining

Following 2D Phos-tag SDS-PAGE, the gels were stained using Pro-Q Diamond phosphoprotein gel stain (Molecular Probes, Inc., Eugene, OR, USA) according to the manufacturer’s protocol, and the stained gel images were recorded using FLA-7000 (Fujifilm Corporation, Tokyo, Japan). The gels were also stained with SYPRO Ruby protein gel stain (Molecular Probes, Inc., Eugene, OR, USA), which detected all proteins, and it was used as a control in Pro-Q Diamond staining.

### Protein identification by mass spectrometry

Protein spots in the 2D Phos-tag SDS-PAGE gel were identified by LC–MS/MS. The spots in the silver-stained 2D gel were cut out using a biopsy punch (diameter: 2 mm; Kai Industries Co., Ltd., Tokyo, Japan). Gel pieces were de-stained with de-staining solution (15 mM potassium ferricyanide and 50 mM sodium thiosulfate) and were washed with Milli-Q water. After dehydration with acetonitrile, the gel pieces were dried under vacuum. The proteins in the gel pieces were reduced (10 mM DTT and 25 mM ammonium hydrogen carbonate reagent solution) at 56 °C for 1 h, and 25 mM ammonium hydrogen carbonate was used for washing. The proteins were alkylated in alkylating solution (55 mM iodoacetamide and 25 mM ammonium hydrogen carbonate) for 45 min. The gel pieces were then washed in 25 mM ammonium hydrogen carbonate and dehydrated with dehydrating solution (50% acetonitrile [ACN] and 25 mM ammonium hydrogen carbonate) two times. After drying under vacuum, the proteins were digested with trypsin at 37 °C overnight. The peptides were extracted from the gel pieces with extraction solution (50% CAN and 5% trifluoroacetic acid [TFA]). The extraction solution containing peptides was completely dried under vacuum. The peptides were then dissolved in Milli-Q:ACN:TFA = 98:2:0.1 and analyzed using HTC PAL (CTC Analytics AG, Zwingen, Switzerland). Raw data files were filtered using Proteome Discoverer software ver. 2.1 (Thermo Fisher Scientific, Waltham, MA, USA).

### Subfractionation of the thylakoid membrane

The thylakoid membrane purified from dark-adapted leaves of 8-week-old plants was subfractionated into grana core, grana margin, and stroma lamella according to Wang et al. (2016) with a slight modification. In brief, the purified thylakoid membrane sample was resuspended with a suspension buffer (100 mM Sorbitol, 50 mM HEPES–NaOH (pH 7.5), and 2 mM MgCl_2_) containing cOmplete Tablets, Mini EDTA-free, EASYpack protease inhibitor cocktail (F. Hoffmann-La Roche AG, Basel, Switzerland). Phosphatase inhibitors (10 mM NaF and 0.5 mM NaVO_3_) were added to the buffer before use. The suspension was diluted to 0.6 mg Chl/mL with the suspension buffer and was incubated in dark at room temperature for 45–60 min. The suspension was gently mixed with an equal volume of 1.6% (w/v) glyco-diosgenin (GDN) that was dissolved in the same suspension buffer. After incubation in dark for 10 min at room temperature, insoluble materials were removed by centrifugation at 1000 × *g* for 1 min at 4 °C. The supernatant was centrifuged with a fixed angle rotor at 40,000 × *g* for 30 min at 4 °C using the Optima L-100 K ultracentrifuge (Beckman Coulter, Inc., Brea, CA, USA) with an open-top polycarbonate tube (10 mL; Beckman Coulter, Inc., Brea, CA, USA) to obtain the pellet of grana core. The supernatant fraction was centrifuged again at 140,000 × *g* for 90 min at 4 °C, resulting in a loose pellet (grana margin) and a tight pellet (stroma lamella). The grana core, grana margin, and stroma lamella pellets were resuspended in the suspension buffer, using fine brush. Protein concentration was measured using the Piece BCA Protein Assay kit (Thermo Fisher Scientific, Waltham, MA, USA).

### Immunoblotting

Phos-tag gel was incubated in SDS-PAGE running buffer containing 10 mM EDTA for 10 min three times to remove metal ion; it was then equilibrated in transfer buffer for 10 min. Subsequently, the gel was electroblotted to the polyvinylidene difluoride (PVDF) membrane (ATTO Corporation, Tokyo, Japan). The PVDF membrane was incubated in 1% (w/v) bovine serum albumin in TBST buffer for 1 h. The membrane was then incubated with the primary antibody in TBST buffer for 1 h. After washing with TBST buffer three times, the membrane was incubated with the secondary antibody Amersham ECL Rabbit IgG and HRP-linked F(ab)_2_ fragment (from donkey) (dilution, 1:10,000; GE Healthcare, Chicago, IL, USA) in TBST buffer for 1 h. To detect immunoblot signals, the membrane was treated with Luminata Crescendo Western HRP Substrate (EMD Millipore Corporation, Burlington, MA, USA) and the chemiluminescence signals was imaged using the Molecular Imager ChemiDoc XRS + imaging system and Image Lab software ver. 4.0.1 (Bio-Rad Laboratories, Inc., Hercules, CA, USA). For immunoblotting of anti-CURT1A, Signal Enhancer HIKARI for Western blotting and ELISA (Nacalai Tesque, Kyoto, Japan) was used instead of TBST buffer in the incubation process with both primary and secondary antibodies; the dilution rate for the secondary antibody was 1:5000. The dilution rate of the primary antibody was follows: anti-CURT1A 1:1000, anti-CP43 1:5000(AS11 1787, Agrisera), anti-D1 1:10,000 (Kato et al. 2012), anti-D2 1:5000 (AS06 146, Agrisera), anti-FtsH2/8 1:5000 (Sakamoto et al. 2003), anti-CP43 1:5000 (AS11 1787, Agrisera), anti-Lhcb2 1:5000 (AS01 003, Agrisera), and anti-Lhcb4 1:5000 (AS04 045, Agrisera).

## Results

### 2D Phos-tag-based detection of protein phosphorylation in the thylakoid membrane

**Fig. 1:**
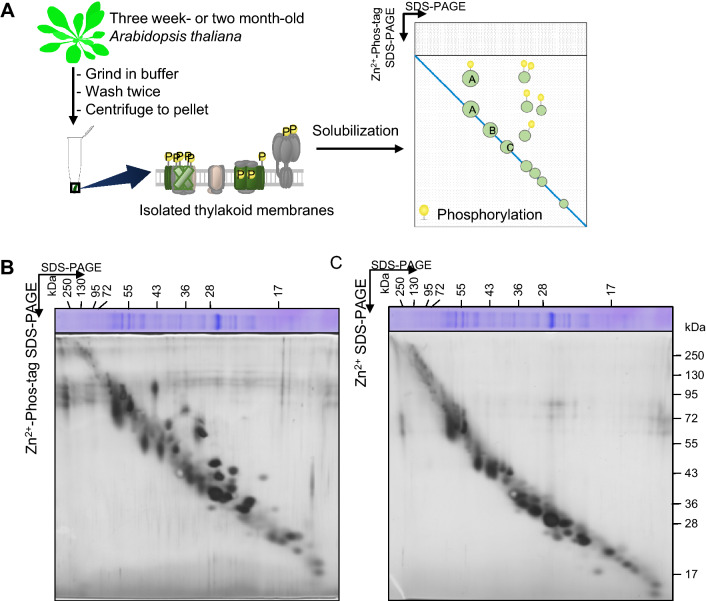
Detection of phosphorylated thylakoid proteins by 2D Phos-tag SDS-PAGE. **a** General outline of 2D Phos-tag SDS-PAGE in this study. Schematic representation of the experiment performed in this study. Phosphorylation of thylakoid membrane proteins was analyzed by 2D Phos-tag SDS-PAGE. Proteins from isolated thylakoid membranes were separated by conventional SDS-PAGE at the first dimension and were separated by Zn^2+^-Phos-tag SDS-PAGE at the second dimension. **b**, **c** Thylakoid proteins extracted from Col leaves were separated by 2D Phos-tag SDS-PAGE [(first dimension was SDS-PAGE/second dimension was Zn^2+^-Phos-tag SDS-PAGE (**b**)] and 2D PAGE [(first dimension was SDS-PAGE/second dimension was Zn^2+^ SDS-PAGE without Phos-tag (**c**)]. All gels were subjected to silver staining. Proteins were equally loaded based on the total chlorophyll content. As the gels did not contain lower molecular weight proteins close to the dye front, the staining pattern in the low molecular weight range appeared slightly different from the gel images in other figures

To detect the phosphorylation of major thylakoid membrane proteins using Phos-tag, we tried to develop 2D Phos-tag SDS-PAGE of the thylakoid membrane. First, thylakoid membrane proteins isolated from *Arabidopsis* ecotype Columbia (Col) were separated in the first dimension by conventional SDS-PAGE and followed by Phos-tag SDS-PAGE in the second dimension (Fig. 1). The 2D Phos-tag SDS-PAGE showed that the mobility of many thylakoid membrane proteins was retarded in the second dimension, implying that numerous thylakoid membrane proteins were phosphorylated (Fig. 1b). To test whether the retarded bands are a result of Phos-tag, Zn^2+^ SDS-PAGE without Phos-tag was performed as the negative control. The result showed that the upward shift of most of the proteins was not observed in the gel without Phos-tag (Fig. 1c). However, the mobility of several proteins seemed to shift slightly upward in the second dimension. This unexpected retardation in these proteins can be attributed to the difference in the gel composition and buffer system used for electrophoresis between Phos-tag SDS-PAGE and conventional Laemmli’s SDS-PAGE. To confirm this possibility, we performed 2D PAGE in which both first and second dimensions were electrophoresed by Zn^2+^ SDS-PAGE without Phos-tag to linearize non-phosphorylated protein spots (Supplementary Fig. S1). Consequently, when the same gel composition and buffer system were used, the thylakoid membrane proteins migrated in a straight line in the gel without Phos-tag, and the phosphorylated proteins were detected as upward-shifted spots in the gel with Phos-tag. Overall, we decided to use 2D Phos-tag SDS-PAGE (Fig. 1); the first dimension was run by conventional SDS-PAGE in further experiments by taking into account the separation of each protein spot.

**Fig. 2:**
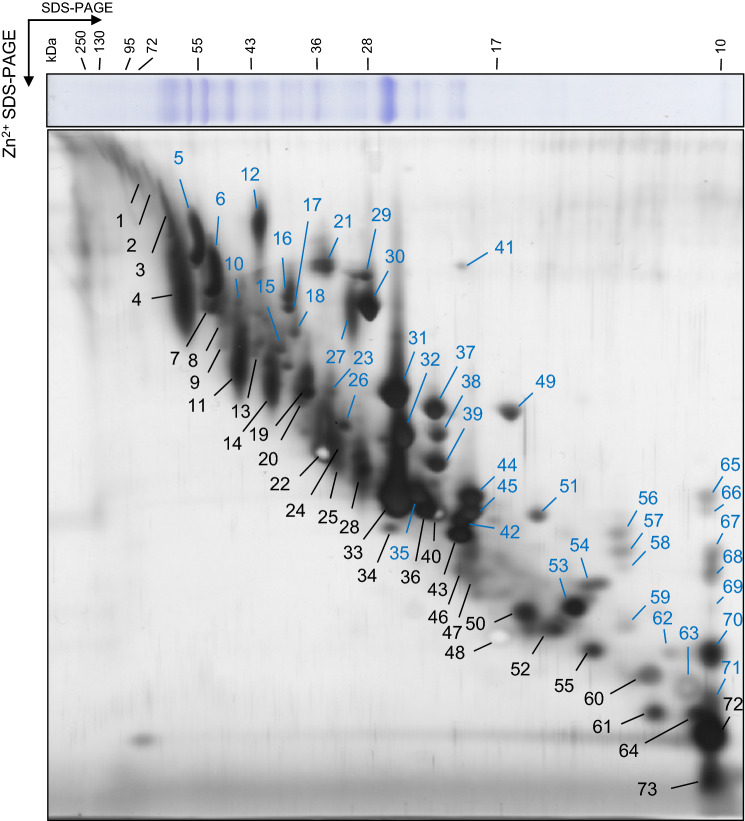
Identification of protein spots detected on 2D Phos-tag SDS-PAGE by LC–MS/MS. The protein spots on the 2D Phos-tag SDS-PAGE in this figure were serially numbered. Blue and black numbers, respectively, denote protein spots shifted and non-shifted upward, compared with the gel without Phos-tag. Identified proteins are listed in Table 1

To identify protein spots in the 2D Phos-tag SDS-PAGE gel, the thylakoid membrane proteins in the gel pieces were digested into peptides with trypsin, and the peptides were analyzed by LC–MS/MS. Seventy-three protein spots in the gel were subjected to peptide identification. Of these, 51 proteins were identified by LC–MS/MS (Fig. 2; Table 1). The results showed that several proteins were detected as spots in different locations, suggesting that specific phosphorylation altered the mobility of proteins in the Phos-tag gel. The protein spots shifted upward; they were numbered as 12, 21, 27, and 31 and were identified as the phosphorylated forms of the PSII core proteins (CP43, D2, and D1) and major antenna proteins, Lhcb1 and Lhcb12. These well-known phosphorylated proteins in the thylakoid membrane indicated that our 2D Phos-tag SDS-PAGE successfully separated the phosphorylated form of thylakoid membrane proteins. We also identified minor antenna proteins Lhcb4.1 (CP29.1) and Lhcb4.2 (CP29.2) in the spots numbered 30. Additionally, Lhcb4.2 was identified in spot 29, whereas Lhcb4.1 was not detected in this spot. A previous study reported that phosphorylated Lhca4 was hardly detectable by conventional immunoblot approach using anti-phosphothreonine antibodies (Ihnatowicz et al. 2008). However, the mobility shift of Lhca4 was reproducibly observed in our 2D Phos-tag SDS-PAGE.

**Table 1:** List of thylakoid membrane proteins identified by LC–MS/MS

#	Accession	Symbol	Sequence	Position	XCorr
1	AT5G50920.1	CLPC1	[R].VLELSLEEAR.[Q]	200–209	3.22
	AT3G48870.1	CLPC2			
	AT3G48870.2				
	AT3G48870.3				
	AT3G48870.4				
2	AT2G41520.1	TPR15	[K].LNISDSR.[V]	260–266	1.99
	AT2G41520.2				
	AT3G47060.1	FTSH7	[-].MTTpTFEFLQPR.[I]	1–11	2.80
3	AT2G30950.1	FTSH2	[K].GVLLIGPPGTGK.[T]	262–273	2.41
	AT5G42270.1	FTSH5	[R].GGQGGAGGPGGLGGPMDFGR.[S]	215–234	4.55
			[R].ELKEISKDEISDALER.[I]	474–489	3.39
			[K].EISKDEISDALER.[I]	477–489	3.35
4	ATCG00350.1	PSAA	[K].DILEAHKGPFTGQGHK.[G]	321–336	3.23
			[R].YNDLLDR.[V]	420–426	2.01
	ATCG00340.1	PSAB	[R].FSQGLAQDPTTR.[R]	8–19	4.09
			[R].TNFGIGHSIK.[D]	293–302	2.94
			[K].DLLEAHIPPGGR.[L]	303–314	3.37
			[R].DYNPEQNEDNVLAR.[M]	397–410	4.43
			[K].DFGYSFPCDGPGR.[G]	552–564	4.05
			[R].TPLANLIR.[W]	685–692	2.71
5*	ATCG00120.1	ATPA	[R].VINALANPIDGR.[G]	108–119	3.68
			[K].ASSVAQVVTSLQER.[G]	203–216	3.97
			[R].EQHTLIIYDDLSK.[Q]	254–266	3.18
			[K].TNKPQFQEIIASTK.[T]	467–480	4.24
6*	ATCG00480.1	ATPB	[R].TNPTTSNPEVSIR.[E]	3–15	4.02
			[R].TNPTTSNPEVSIREK.[K]	3–17	3.60
			[K].MPNIYNALVVK.[G]	40–50	3.98
			[R].AVAMSATEGLKR.[G]	76–87	3.27
			[R].IFNVLGEPVDNLGPVDTR.[T]	110–127	4.88
			[K].IGLFGGAGVGK.[T]	168–178	2.93
			[K].TVLIMELINNIAK.[A]	179–191	4.32
			[R].TREGNDLYMEMK.[E]	206–217	3.70
			[K].ESGVINEQNLAESK.[V]	218–231	4.36
			[K].VALVYGQMNEPPGAR.[M]	232–246	4.76
			[R].FVQAGSEVSALLGR.[M]	278–291	5.01
			[R].MPSAVGYQPTLSTEMGTLQER.[I]	292–312	4.52
			[K].GIYPAVDPLDSTSTMLQPR.[I]	360–378	4.71
			[R].IVGEEHYETAQQVK.[Q]	379–392	4.84
			[R].IVGEEHYETAQQVKQTLQR.[Y]	379–397	4.52
			[K].ATNLEMESK.[L]	487–495	2.91
7	ATCG00490.1	RBCL	[K].TFQGPPHGIQVER.[D]	147–159	3.68
			[R].LSGGDHIHAGTVVGK.[L]	320–334	4.13
8	AT4G20360.1	RABE1B	[K].KPHVNIGTIGHVDHGK.[T]	77–92	3.41
			[K].KYDEIDAAPEER.[A]	113–124	4.38
			[K].VGETVDLVGLR.[E]	316–326	3.63
			[R].TTDVTGKVTK.[I]	412–421	2.59
			[K].IMNDKDEESK.[M]	422–431	2.84
	AT2G39730.1	RCA	[R].KFVESLGVEK.[I]	359–368	2.60
	AT2G39730.2				
	AT2G39730.3				
9	n.d				
10*	n.d				
11	ATCG00680.1	CP47	[R].VHTVVLNDPGR.[L]	8–18	3.22
			[K].LAFYDYIGNNPAK.[G]	309–321	4.54
			[R].RAQLGEIFELDR.[A]	423–434	3.50
			[R].AQLGEIFELDR.[A]	424–434	3.90
12*	ATCG00280.1	CP43	[R].LINLSGK.[L]	42–48	2.19
			[R].LGANVGSAQGPTGLGK.[Y]	324–339	4.63
			[R].SPTGEVIFGGETMR.[F]	344–357	4.76
			[R].AAAAGFEK.[G]	450–457	2.12
13	n.d				
14	ATCG00280.1	CP43	[R].LGANVGSAQGPTGLGK.[Y]	324–339	4.50
			[R].SPTGEVIFGGETMR.[F]	344–357	4.13
			[R].AAAAGFEK.[G]	450–457	1.81
15*	AT2G35490.1	PAP3	[R].SPSRIEVSFK.[E]	268–277	2.95
16*	AT2G21330.1	FBA1	[R].LASIGLENTEANR.[Q]	84–96	3.95
	AT2G21330.3		[K].YTGEGESEEAK.[E]	379–389	3.32
	AT4G38970.1	FBA2	[K].RLDSIGLENTEANR.[Q]	82–95	4.96
			[R].LDSIGLENTEANR.[Q]	83–95	4.31
			[K].YTGEGESEEAK.[E]	378–388	3.32
17*	AT4G38970.1	FBA2	[K].RLDSIGLENTEANR.[Q]	82–95	4.96
			[R].LDSIGLENTEANR.[Q]	83–95	4.31
			[K].YTGEGESEEAK.[E]	378–388	3.32
18*	AT5G66190.1	FNR1	[R].LVYTNDGGEIVK.[G]	164–175	3.17
19	AT4G04640.1	ATPC1	[R].DRIDSVKNTQK.[I]	58–68	2.75
			[K].KVALVVVTGDR.[G]	126–136	3.98
			[R].RPYIPVDKYLEAGTLPTAK.[E]	180–198	4.66
			[K].YLEAGTLPTAK.[E]	188–198	3.26
			[K].SEPVIHTLLPLSPK.[G]	232–245	3.99
			[R].ALQESLASELAAR.[M]	316–328	2.95
20	AT1G20020.1	FNR2	[K].VSKKNEEGVIVNR.[Y]	74–86	3.64
	AT1G20020.3		[K].KNEEGVIVNR.[Y]	77–86	2.81
			[K].NEEGVIVNR.[Y]	78–86	3.10
21*	ATCG00270.1	D2	[R].AYDFVSQEIR.[A]	296–305	4.4
22	AT3G50820.1	PSBO2	[R].GGSTGYDNAVALPAGGR.[G]	247–263	4.06
			[R].GDEEELSKENVK.[N]	264–275	3.03
			[K].NTAASVGEITLK.[IV]	276–287	3.67
23*	n.d				
24	AT1G08065.1	ACA5	[R].FIGSLpTTPPCSENVIWTISK.[E]	210–229	3.37
25	ATCG00270.1	D2	[R].AYDFVSQEIR.[A]	296–305	4.57
26*	AT4G04020.1	PGL35	[K].AIESVEETER.[L]	84–93	3.43
27*	ATCG00020.1	D1	[-].MpTAILERR.[E]	1–8	2.77
			[R].ETTENESANEGYR.[F]	226–238	3.21
			[R].ANLGMEVMHER.[N]	324–334	3.48
28	ATCG00020.1	D1	[R].FCNWITSTENR.[L]	17–27	3.30
			[R].ETTENESANEGYR.[F]	226–238	4.10
			[R].ANLGMEVMHER.[N]	324–334	3.52
29*	AT3G08940.2	LHCB4.2	[K].NLYGEVIGTR.[T]	101–110	3.41
			[K].AQLQLAEIK.[H]	233–241	2.78
30*	AT5G01530.1	LHCB4.1	[K].NLAGDVIGTRTEAADAK.[S]	104–120	4.30
			[R].FRECELIHGR.[W]	136–145	3.53
			[R].NAELDSEK.[R]	207–214	2.05
			[K].FFDPLGLAADPEK.[T]	222–234	4.37
			[K].TAQLQLAEIK.[H]	235–244	3.88
			[K].TAQLQLAEIKHAR.[L]	235–247	3.82
	AT3G08940.2	LHCB4.2	[R].FRECELIHGR.[W]	133–142	3.53
			[R].NAELDSEK.[R]	204–211	2.05
			[K].FFDPLGLASDPVKK.[A]	219–232	4.43
			[K].KAQLQLAEIK.[H]	232–241	3.55
			[K].AQLQLAEIK.[H]	233–241	3.32
31*	AT1G29920.1	LHCB1.1	[RK].NRELEVIHSR.[W]	95–104	3.48
	AT1G29910.1	LHCB1.2			
	AT1G29930.1	LHCB1.3			
	AT2G34430.1	LHCB1.4			
	AT2G34420.1	LHCB1.5			
	AT2G05100.1	LHCB2.1			
	AT2G05070.1	LHCB2.2			
	AT3G27690.1	LHCB2.3			
32*	AT4G10340.1	LHCB5	[K].SKAVSETSDELAK.[W]	49–61	4.41
			[K].AVSETSDELAK.[W]	51–61	3.38
			[K].YQAFELIHAR.[W]	110–119	3.27
			[R].ITNGLDFEDK.[L]	190–199	3.63
			[K].LHPGGPFDPLGLAK.[D]	200–213	2.98
			[K].DPEQGALLK.[V]	214–222	3.33
33	AT1G29920.1	LHCB1.1	[RK].NRELEVIHSR.[W]	95–104	3.65
	AT1G29910.1	LHCB1.2			
	AT1G29930.1	LHCB1.3			
	AT2G34430.1	LHCB1.4			
	AT2G34420.1	LHCB1.5			
	AT2G05100.1	LHCB2.1			
	AT2G05070.1	LHCB2.2			
	AT3G27690.1	LHCB2.3			
34	AT1G54780.1	TLP18.3	[K].KLLSDLEYR.[K]	117–125	3.01
			[K].ADAFEYADQVLEK.[W]	142–154	3.10
			[K].TKEETDEKR.[G]	248–256	3.05
35*	AT3G54890.1	LHCA1	[R].YKESELIHCR.[W]	83–92	3.27
36	n.d				
37*	AT1G61520.1	LHCA3	[R].RLQDWYNPGSMGK.[Q]	170–182	3.40
			[R].LQDWYNPGSMGK.[Q]	171–182	3.65
38*	n.d				
39*	n.d				
40	n.d				
41*	AT3G47470.1	LHCA4	[K].NPGSVNQDPIFK.[Q]	163–174	3.59
42*	AT4G09650.1	ATPD	[K].LTDTQLAEVR.[S]	149–158	3.78
43	AT1G44575.1	PSBS	[K].FITDDGEES.[-]	257–265	2.06
	AT3G47470.1	LHCA4	[K].NPGSVNQDPIFK.[Q]	163–174	3.79
44*	AT1G15820.1	LHCB6	[K].NRDGVYEPDFEKLER.[L]	209–223	4.02
			[R].DGVYEPDFEKLER.[L]	211–223	3.54
			[R].LKLAEIKHSR.[L]	224–233	3.54
45*	AT1G15820.1	LHCB6	[R].FFDPLGLAGK.[N]	199–208	3.01
			[R].DGVYEPDFEKLER.[L]	211–223	3.54
46	AT4G02770.1	PSAD1	[R].AEKTDSSAAAAAAPATK.[E]	45–61	4.78
			[K].TDSSAAAAAAPATK.[E]	48–61	3.28
			[R].KEQCLALGTR.[L]	132–141	3.27
47	AT1G03130.1	PSAD-2	[R].AEKTESSSAAPAVK.[E]	44–57	3.26
			[R].KEQCLALGTR.[L]	128–137	3.44
			[K].DGVYPEK.[A]	164–170	1.90
			[R].SIGKNVSPIEVK.[F]	184–195	3.48
48	n.d				
49*	AT4G32260.1	ATPG	[K].LASVKDTSTEVK.[E]	125–136	2.93
			[K].ELDEQAAAVMR.[A]	137–147	2.68
			[R].AEIAAALNK.[M]	151–159	2.25
			[K].KETQVEVEEK.[L]	162–171	3.46
			[R].KKVEEELK.[E]	177–184	2.64
50	AT4G21280.1	PSBQ1	[K].VGPPPAPSGGLPGTDNSDQAR.[D]	82–102	3.14
			[R].DFALALK.[D]	103–109	2.36
			[R].FYLQPLPPTEAAAR.[A]	112–125	3.16
			[K].DIINVKPLIDR.[K]	132–142	3.34
51*	AT3G27830.1	RPL12A	[K].EGITKDEAEEAK.[K]	167–178	2.93
	AT3G27850.1	RPL12C	[K].EGITKDEAEEAKK.[T]	167–179	3.38
52	ATCG00130.1	ATPF	[K].GVLNDLLDNR.[K]	47–56	3.50
			[R].EGAIQQLENAR.[A]	72–82	3.67
			[R].LRNVETEADKFR.[V]	85–96	3.92
	AT1G31330.1	PSAF	[K].KLESSLK.[L]	91–97	2.14
			[R].RFDNYGK.[Y]	119–125	2.80
			[R].SYLIAISGEK.[K]	170–179	3.42
			[K].EIIIDVPLASR.[I]	185–195	3.66
53*	AT4G28750.1	PSAE-1	[K].NVGSVVAVDQDPK.[T]	102–114	4.49
	AT2G20260.1	PSAE-2	[K].TRYPVVVR.[F]	115–122	2.94
54*	n.d				
55	ATCG00470.1	ATPE	[R].VEALNTI.[-]	126–132	2.19
56*	n.d				
57*	n.d				
58*	n.d				
59*	n.d				
60	AT3G16140.1	PSAH1	[K].RGPQEPPKLGPR.[G]	131–142	3.31
	AT1G52230.1	PSAH2	[R].GPQEPPKLGPR.[G]	132–142	2.84
61	n.d				
62*	n.d				
63*	AT1G79040.1	PSBR	[K].TDKPFGINGSMDLR.[D]	49–62	3.08
64	n.d				
65*	n.d				
66*	n.d				
67*	n.d				
68*	n.d				
69*	n.d				
70*	ATCG00580.1	PSBE	[R].SFADIITSIR.[Y]	9–18	4
71*	AT3G47070.1	TSP9	[K].KVDEKEGTTTGGR.[G]	57–69	3.86
72	AT1G55670.1	PSAG	[R].ENVAKQGLPEQNGK.[T]	89–102	4.24
			[K].THFEAGDDR.[A]	103–111	3.12
			[K].THFEAGDDRAK.[E]	103–113	3.64
			[R].AKEYVSLLK.[S]	112–120	2.73
	AT1G30380.1	PSAK	[R].FGLAPSANR.[K]	70–78	2.29
			[R].KATAGLRLEAR.[D]	79–89	3.10
73	n.d				

The proteins in gel pieces from 2D Phos-tag SDS-PAGE were proteolyzed to peptides with trypsin. Detected data were analyzed by Proteome Discoverer 2.1 software. Spot numbers “#” indicate the spots in Fig. 2. Asterisk means the spot shifted in Fig. 2. Small letter “p” in sequence refers to the phosphorylated amino acid just behind the letter. n.d., not detected. The Xcorr values calculated by using the SEQUEST algorithm (Eng et al. 1994) are shown

**Fig. 3:**
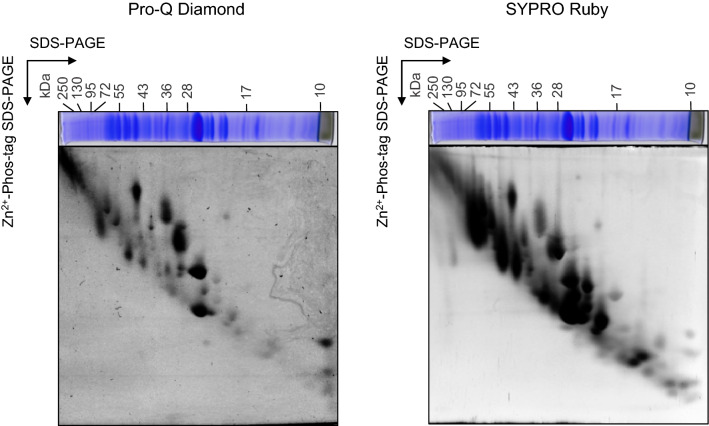
Detection of phosphorylated thylakoid proteins on 2D Phos-tag SDS-PAGE gel by Pro-Q diamond staining. The thylakoid proteins extracted from Col leaves were separated by 2D Phos-tag SDS-PAGE (the first dimension was SDS-PAGE/second dimension was Zn^2+^-Phos-tag SDS-PAGE). The gel was subjected to Pro-Q diamond staining or SYPRO Ruby staining. For Pro-Q diamond staining, phosphoproteins were detected specifically. SYPRO Ruby staining was utilized as a control experiment of Pro-Q diamond staining. Proteins were equally loaded based on the total chlorophyll content

To verify that the mobility shift of proteins in the second dimension of 2D Phos-tag SDS-PAGE-reflected protein phosphorylation, we stained the 2D Phos-tag SDS-PAGE gel with Pro-Q diamond, which is a reagent for specifically staining phosphoproteins (Fig. 3). As expected, the Pro-Q diamond signal was detected in the protein spots that shifted upward (e.g., PSII core and LHCII proteins). On the contrary, a spot of protein of molecular weight less than 10 kDa exhibited a strong signal. Protein spot 70, identified as PsbE by LC–MS/MS, is located around this region. However, PsbE has not been reported as a phosphoprotein. Another PSII subunit PsbH, reported as phosphorylated, might migrate to a similar position, whereas our LC–MS/MS analysis could not identify PsbH in the gel. Contrarily, a small number of protein spots showed retarded mobility in the gel and were not stained by Pro-Q diamond staining, suggesting that the mobility shift of these proteins was not due to phosphorylation (false positive) or that the phosphorylation of these proteins was not effectively detected by Pro-Q diamond staining. Pro-Q diamond also appeared to stain several proteins, which did not exhibit mobility shift. A previous study reported a case of no mobility shift of phosphorylated proteins in Mn^2+^-Phos-tag SDS-PAGE (Kinoshita et al. 2008). This result suggests the position of phosphorylated residues in the proteins that influence the mobility of proteins in Phos-tag SDS-PAGE.

### Protein phosphorylation in kinase mutants

**Fig. 4:**
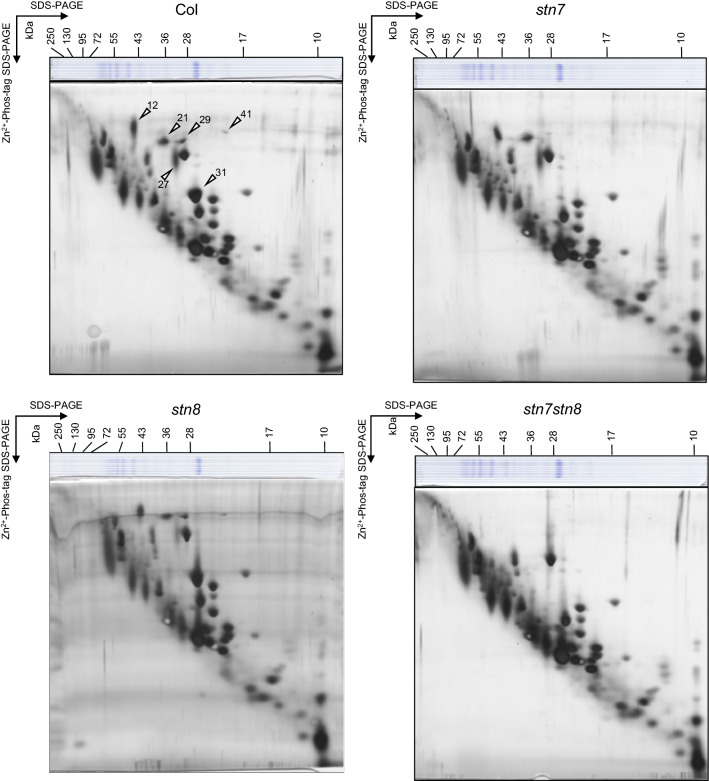
Comparison of phosphorylated thylakoid in the kinase mutants by 2D Phos-tag SDS-PAGE. Thylakoid membrane proteins in Col, *stn7*, s*tn8*, and *stn7stn8* grown under growth light (GL, 160 μmol photons m^−2^ s^−1^) were separated by 2D Phos-tag SDS-PAGE. All gels were subjected to silver staining. Proteins were equally loaded based on the total chlorophyll content. The white arrowheads indicate the spots reduced in the mutants

To evaluate STN7- or STN8-dependent protein phosphorylation in the 2D Phos-tag SDS-PAGE, we investigated the phosphorylated status of thylakoid membrane proteins in the *stn7*, *stn8*, and *stn7stn8* mutants. The results demonstrated a substantial reduction in protein spots, identified as Lhcb1, Lhcb2, and Lhcb4.2 (spots 31 and 29), and the elimination of protein spots, identified as Lhca4 (spot 41), in *stn7* thylakoid proteins (Fig. 4 and Supplementary Fig. S2). This result was consistent with that of previous studies, which reported that the phosphorylation of Lhcb1, Lhcb2, Lhcb4.2, and Lhca4 depends on STN7 (Bellafiore et al. 2005; Tikkanen et al. 2006; Ihnatowicz et al. 2008).

On the contrary, the 2D Phos-tag SDS-PAGE of *stn8* thylakoid proteins showed that STN8 deficiency only led to a slight decrease in a mobility-retarded spot, identified as D1 (spot 27) (Fig. 4 and Supplementary Fig. S2). A previous study reported that the effect of STN8 deficiency on the phosphorylation of PSII core proteins manifests under high-intensity light conditions (Bonardi et al. 2005). Thus, we further analyzed the phosphorylation status of thylakoid membrane proteins in *stn8* after exposure to high-intensity light (HL, 1200 μmol photons m^−2^ s^−1^ for 2 h) (Supplementary Fig. S3). The result showed that the substantial reduction in three spots, identified as PSII core proteins (CP43, D2, and D1; spots 12, 21, and 27, respectively), was caused in the *stn8* mutant under high-light conditions. Subsequently, the phosphoproteins in the thylakoid membrane of *stn7stn8* were collectively analyzed by 2D Phos-tag SDS-PAGE (Fig. 4 and Supplementary Fig. S2), and consequently, all mobility-retarded spots, identified as LHC (Lhcb1, Lhcb2, Lhcb4.2, and Lhca4) and PSII core proteins (CP43, D2, and D1), were completely eliminated. These results further confirmed that our 2D Phos-tag SDS-PAGE was successfully performed, and the phosphorylation status of these proteins could be evaluated by Phos-tag SDS-PAGE. In contrast to the substantial reduction in the phosphorylation in LHC and PSII core proteins, other protein spots that shifted upward were not affected even in the *stn7stn8* double mutant.

### Phos-tag-based assay of protein phosphorylation in the thylakoid membrane subfractions

**Fig. 5:**
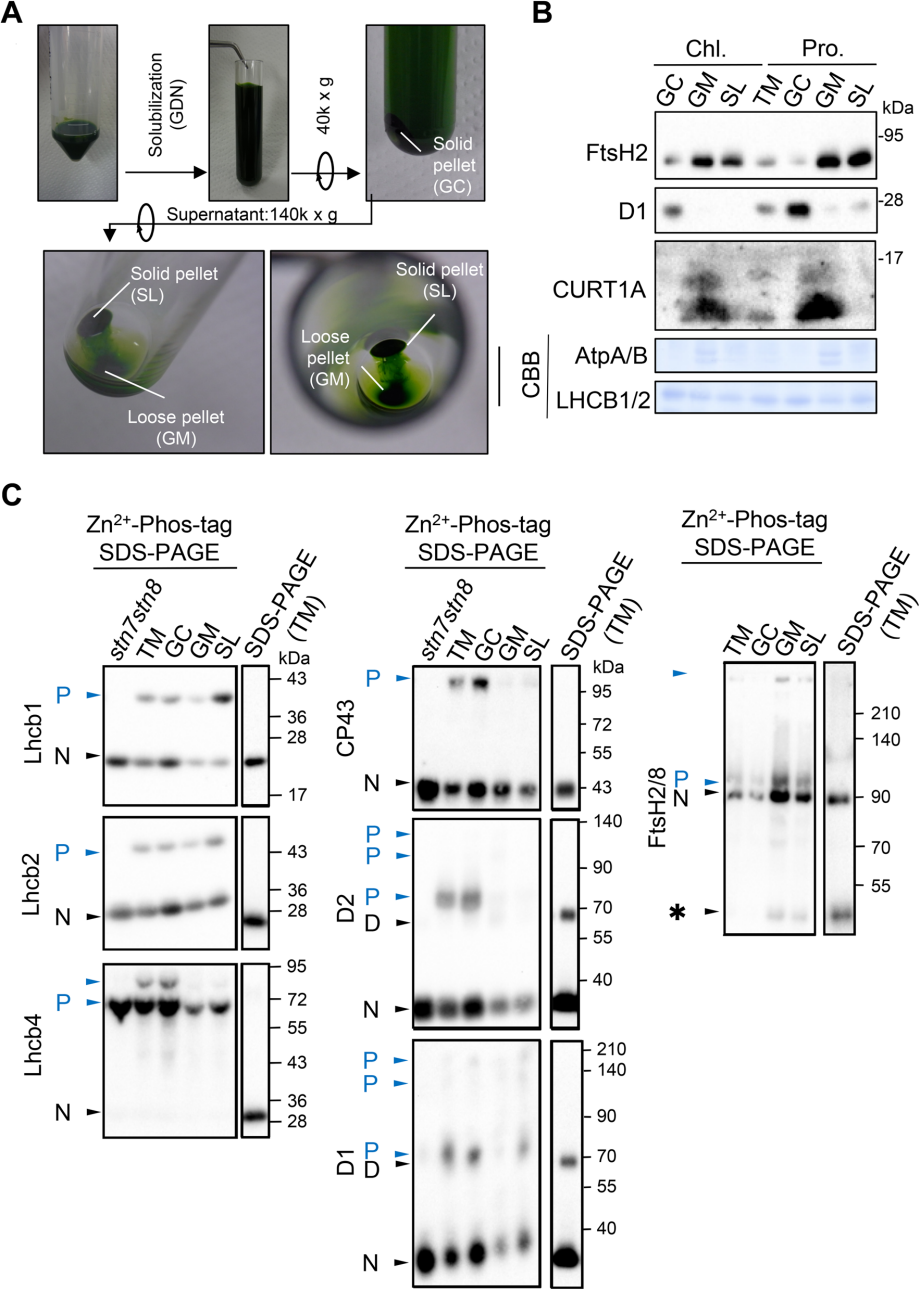
Detection of protein phosphorylation in the thylakoid membrane after subfractionation. **a** Thylakoid membranes (TM) were separated into grana core (GC), grana margin (GM), and stroma lamella (SL) with glyco-diosgenin (GDN). **b** Protein accumulation in each subfraction. Immunoblotting of marker proteins for thylakoid subfractionation (D1, grana core; CURT1A, grana margin; FtsH2, stroma-exposed thylakoid), CBB-stained ATP A/B, and Lhcb proteins. Proteins were equally loaded based on the total chlorophyll contents (Chl.) or protein concentration (Pro.). **c** Immunoblotting of thylakoid proteins in each subfraction separated by Phos-tag SDS-PAGE. The phosphorylation of LHCII, PSII core proteins, and FtsH protein was detected. Thylakoid membrane proteins in *stn7stn8* were applied as a negative control of phosphorylation. P, phosphorylated; N, non-phosphorylated; D, dimer; asterisk, non-specific band. Proteins were equally loaded based on the total protein concentration

We analyzed the protein phosphorylation status in the thylakoid membrane subfractions: grana core, grana margin, and stroma lamella. The grana cores are stacked thylakoid membrane areas and are connected at grana margins to non-appressed stroma thylakoids, called stroma lamella. The PSII-LHCII supercomplexes are enriched in the grana cores in contrast to the PSI complex and are mainly localized in the stroma lamella. To characterize protein phosphorylation in these subfractions, we solubilized the thylakoid membranes with glyco-diosgenin (GDN) as a substitute for the commonly used digitonin (Fig. 5a). The results showed that Lhcb1 and Lhcb2 were enriched in the grana cores, whereas the ATP synthase complexes were detected in the stroma-exposed thylakoids, grana margins, and stroma lamellae. These results were consistent with those of Wang et al. (2016), who used digitonin for solubilization. The chlorophyll a/b ratio in the grana cores and margins was close to the optimal values (grana core, 2.0–2.4; grana margin, < 3.5), whereas the ratio in the stroma lamella still remained less than 4.0. Furthermore, we investigated the abundance of the following marker proteins: PSII reaction center protein D1 in the grana core and CURT1A in the grana margin (Fig. 5b). CURT1A is also oligomerized with other CURT1s and forms curvature domains in the thylakoid membrane (grana margin) for fine-tuning photosynthesis (Armbruster et al. 2013; Pribil et al. 2018). As shown in Fig. 5, D1 and CURT1A were enriched in the grana cores and grana margins, respectively. These results are consistent with those of previous studies using digitonin (Wang et al. 2016), suggesting that solubilization with GDN can successfully subfractionate the thylakoid membrane.

**Fig. 6:**
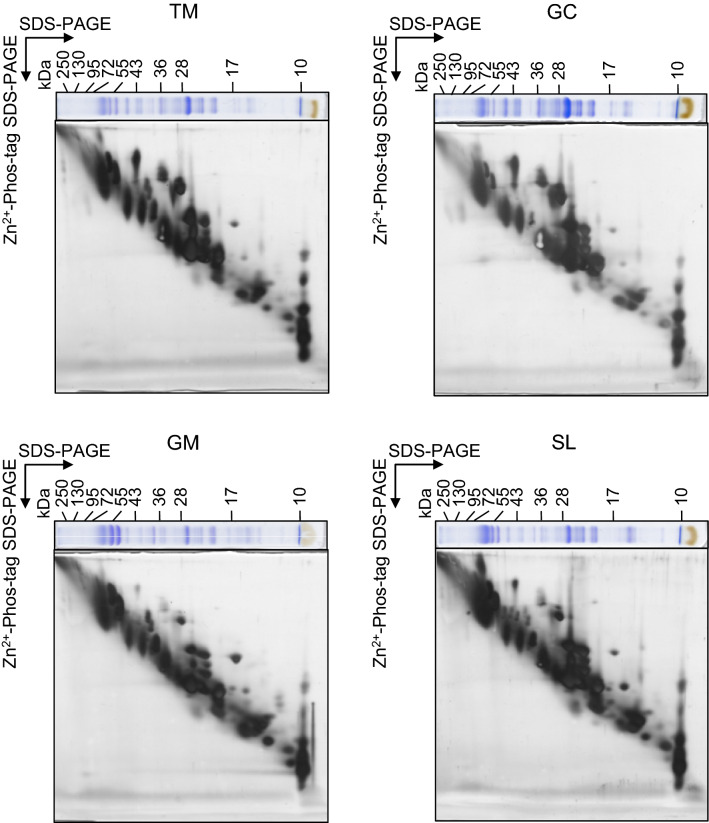
Detection of protein phosphorylation in different thylakoid subfractions by 2D Phos-tag SDS-PAGE. Thylakoid membranes (TMs) were subfractionated into grana cores (GC), grana margins (GM), and stroma lamella (SL) with detergent glyco-diosgenin (GDN). Proteins were equally loaded based on the total protein concentration (Pro)

The thylakoid membrane subfractions were normalized on the basis of protein content, as loading based on the chlorophyll content tends to be strongly affected by LHC abundance (Fig. 5b). The 2D Phos-tag assay of thylakoid subfraction proteins showed the phosphorylation of PSII core subunits and LHCII proteins in the grana cores and the protein spots representing the phosphorylated isoforms of these proteins decreased in the grana margins and stromal lamellae (Fig. 6). Although we expected to find additional protein spots in the subfractions, no additional proteins were detected compared to the total thylakoid membrane. This may be because photosynthetic-related proteins, which are the primary proteins occupying the thylakoid membrane protein, may mask minor protein signals even in the subfractions. On the contrary, the different phosphorylation status detected in the thylakoid subfractions enabled us to verify the importance of protein phosphorylation among the three thylakoid subfractions. Thus, we further analyzed major phosphorylated proteins in the thylakoid subfractions by Phos-tag SDS-PAGE and immunoblotting.

In *Arabidopsis*, the major Lhcb proteins, Lhcb1, Lhcb2, and Lhcb3, are composed of trimers. Among them, the phosphorylation of Lhcb1 and Lhcb2 contributes to state transition, whereas Lhcb3 lacks an N-terminal phosphorylation site (Damkjaer et al. 2009). The immunoblotting results of the Lhcb1 isoforms showed that the phosphorylated form was present in the stroma lamellae, whereas the non-phosphorylated form was predominant over the phosphorylated forms in the grana cores (Fig. 5c). The phosphorylation of Lhcb2 isoforms showed a similar pattern in the immunoblot, but the phosphorylated form in the stroma lamellae was suppressed compared with that of Lhcb1. Furthermore, the immunoblotting analysis of the minor LHCII antenna complex proteins Lhcb4 (CP29) isoforms showed two mobility-shifted bands. Intriguingly, the signal of Lhcb4, localized on the top of the gel, was preferentially accumulated in the grana cores, whereas a second band from the top, which was identified as Lhcb4.1 and Lhcb4.2, was detected in all subfractions. Additionally, a faint band of non-phosphorylated Lhcb4 was detectable at the bottom of the gel (Fig. 5c). On the contrary, the PSII core proteins, D1, D2, and CP43, were enriched in the grana cores and the phosphorylated forms of these proteins were also detected in the grana cores. In contrast, the phosphorylated forms decreased in the grana margins and stroma lamellae, except for D1 in the stroma lamellae (Fig. 5c). We noted that the aggregation of the D1-D2 dimer was observed in this experimental condition, but the phosphorylated isoforms were distinguished from the non-phosphorylated forms by comparing the signals with those detected in *stn7stn8*. As the phosphorylation of FtsH metalloprotease has been reported by previous studies (Stael et al. 2012; Kato and Sakamoto 2019), we also tested the phosphorylation ratio of FtsH, which is essential for D1 degradation in the thylakoid membrane (Sakamoto et al. 2003; Yu et al. 2004; Zaltsman et al. 2005; Kato and Sakamoto 2009). However, it did not seem to differ in the three thylakoid subfractions, suggesting that the phosphorylation of FtsH is unlike that of LHCII and the PS II core and that it is not associated with the membrane structure.

## Discussion

### Detecting protein phosphorylation in the thylakoid membrane using the Phos-tag approach

Immunoblot analysis with anti-phosphothreonine antibodies is commonly used in studies on protein phosphorylation in the thylakoid membrane. Additionally, studies using phosphoproteomic approaches have demonstrated that numerous proteins in the thylakoid membrane are phosphorylated (Grieco et al. 2016). Mass spectrometry allows high-throughput detection of phosphorylated proteins with high sensitivity. However, this approach still has several limitations. First, enrichment of phosphopeptides before mass spectrometric analysis is required to detect phosphorylated proteins, resulting in large amounts of materials necessary for the analysis. Moreover, the enrichment of phosphopeptides causes difficulty in quantifying the phosphorylation stoichiometry of proteins, and this is an important factor to elucidate the role of protein phosphorylation in photosynthetic components under different light regimes. Second, trypsin digestion of proteins generates peptides of various lengths; too short or large peptides cannot be uniquely mapped to a protein, and this may hamper precise detection of phosphorylated amino acid residues. On the contrary, Phos-tag technology is a relatively simple method. Although immunoblot analysis using anti-phospho-antibodies can detect only major phosphorylated proteins, Phos-tag SDS-PAGE combined with specific antibodies is broadly applicable to the characterization of several phosphorylated proteins. In this case, the sensitivity of target proteins in Phos-tag SDS-PAGE depends on the reactivity of specific antibodies; the non-specific reaction of antibodies occasionally causes difficulty in interpreting the phosphorylated status of the target protein. It is noteworthy that Phos-tag SDS-PAGE enables to quantify the phosphorylated forms relative to non-phosphorylated protein and to distinguish different phosphorylated forms of the same protein.

To analyze protein phosphorylation in the thylakoid membrane, the Phos-tag approach has been used to characterize individual proteins (Crepin and Caffarri 2015; Longoni et al. 2015). Longoni et al. (2015) combined Phos-tag SDS-PAGE with blue native-PAGE to analyze the phosphorylated status of LHCII. In contrast, we performed a comprehensive phosphorylation analysis of thylakoid membrane proteins using established 2D Phos-tag SDS-PAGE. Our analysis showed that 32 potentially phosphorylated proteins were shifted upward in the second-dimension electrophoresis using Phos-tag (Figs. 1 and 2, and Table 1), suggesting the wide applicability of the Phos-tag analysis to further study protein phosphorylation in the thylakoid membranes. On the other hand, in this study, we routinely stained gels using silver staining, which is a sensitive method to visualize protein spots. It is known that the linear dynamic range of silver staining is relatively narrow. Therefore, we did not attempt to quantify the intensities of spots, and we only compared them between gels when the staining was not saturated. Further application of combined 2D Phos-tag PAGE with quantitative protein staining techniques might provide further analysis of the ratio of phosphorylated/non-phosphorylated forms.

The majority of the proteins detected as spots in different locations were the PSII core proteins (CP43, D2, and D1) and LHCII proteins (Lhcb1, Lhcb2, and Lhcb4) (Figs. 1 and 2, and Table 1), as reported previously (Bonardi et al. 2005). We also identified several proteins, the LHCII protein (Lhcb6), LHCI protein (Lhca4), and fructose-bisphosphate aldolase 2 (FBA2), which were detected as possible phosphorylated and non-phosphorylated forms. The 2D Phos-tag SDS-PAGE analysis using STN7 and STN8 kinase mutants (*stn7*, *stn8*, and *stn7stn8*) demonstrated that the kinase activities were mainly responsible for the phosphorylation of only the PSII core and LHCII proteins and not of other proteins (Fig. 4). This result is consistent with those of recent comparative phosphoproteomic studies demonstrating that only a small portion of phosphorylation was STN7/STN8-dependent (Reiland et al. 2011; Ingelsson and Vener 2012; Schönberg et al. 2017), suggesting that the phosphorylation of proteins, except the PSII core and LHCII proteins, occurs independent of these kinase functions. Furthermore, our analysis in the different subfractions of thylakoid membranes showed that the phosphorylation of these proteins is involved in the regulation of localization and function of photosynthetic protein complexes (Fig. 5).

### Analysis of phosphorylation of individual photosynthesis-related proteins

The major proteins that were found to exhibit substantial mobility shift in 2D Phos-tag SDS-PAGE were major LHCII antenna proteins (Lhcb1 and Lhcb2). These proteins are abundant in the thylakoid membrane and are reported to be phosphorylated by STN7 (Bellafiore et al. 2005; Bonardi et al. 2005) and dephosphorylated by PPH1/TAP38 (Shapiguzov et al. 2010; Pribil et al. 2010; Samol et al. 2012). Our 2D Phos-tag results, which showed different phosphorylation levels of these LHCII proteins under light conditions, were consistent with those of previous studies, suggesting that the Phos-tag analysis was successfully performed. Furthermore, the analysis of the thylakoid subfractions revealed that both phosphorylated Lhcb1 and Lhcb2 accumulated primarily in the stroma lamellae rather than in other subfractions. Intriguingly, the phosphorylated form of Lhcb1 was abundant in the stroma lamellae, whereas the phosphorylated form of Lhcb2 was less than the non-phosphorylated form (Fig. 5c). The phosphorylation of Lhcb1 appears to assist the PSII-LHCII supercomplexes to migrate from the appressed grana cores to the stroma-exposed thylakoid membrane, where the PSI is enriched (Grieco et al. 2015; Rantala et al. 2017). Thus, the high phosphorylation rate of Lhcb1 in the stroma lamellae could be explained by the relocation of the PSII-LHCII supercomplexes; the phosphorylation might prevent the complex from migrating back into the grana cores. On the contrary, the phosphorylation of Lhcb2 is likely important for the formation of the PSI-LHCII complex in state 2 of the state transition (Longoni et al. 2015). The different phosphorylation levels between Lhcb1 and Lhcb2 might represent individual states of relocation of the PSII-LHCII complex and the formation of the PSI-LHCII state transition complex.

Besides major LHCII proteins, we found the mobility shift of Lhcb4 (spots 29 and 30), a minor LHCII protein. Previous studies have reported the phosphorylation of Thr-6 in Lhcb4.2 (Tikkanen et al. 2006; Fristedt and Vener 2011). This phosphorylation is undetectable in *stn7stn8*. Contrarily, the phosphorylation at Thr-72 or Thr-74 in Lhcb4.1 and Thr-78 or Thr-80 in Lhcb4.2 has also been reported (Tikkanen et al. 2006; Fristedt and Vener 2011). Fristedt and Vener (2011), based on the mass spectrometry analysis, reported that these phosphorylations are STN7 dependent and likely contribute to the disassembly of the PSII supercomplexes. We observed that the majority of Lhcb4 proteins were constitutively shifted in the Phos-tag gel even in *stn7stn8* (Fig. 4), suggesting that unidentified major kinase-independent phosphorylation occurred in Lhcb4. Further investigations are necessary to elucidate the phosphorylation state of Lhcb4.

Another set of major proteins, namely, the PSII core proteins, CP43, D2, and D1, were clearly shifted by Phos-tag application. The results in this study were consistent with those of previous studies, which reported that phosphorylation by STN8 kinase cooperates with STN7 (Bonardi et al. 2005; Tikkanen et al. 2008; Fristedt et al. 2009). Intriguingly, further immunoblotting in the thylakoid subfractions revealed that almost all phosphorylated forms of CP43 and D2 accumulated in the grana core, yet D1 was phosphorylated even in the stroma lamellae (Fig. 5). In our previous study, we suggested the fine-tuning of D1 degradation by protein phosphorylation in PSII repair; D1 phosphorylation in the N-terminal of D1 prevents the undesirable cleavage of D1 by Deg endoproteases that ultimately leads to the generation of potentially toxic D1 cleavage fragments (Kato and Sakamoto 2014). Based on this study, we interpreted that the higher phosphorylation ratio of D1 in the stroma lamellae was associated with the D1 degradation activity. Contrarily, the phosphorylation of other PSII core proteins, D2 and CP43, would be appropriately dephosphorylated by phosphatase when damaged PSII complex migrates from the grana cores to stroma-exposed thylakoid membranes. We also assessed the phosphorylation status of FtsH, an essential protease for damaged D1 degradation in the PSII repair cycle. FtsH was found to be most abundant in the grana margins, but the ratio of phosphorylated to non-phosphorylated forms of FtsH did not appear to be substantially different among the three subfractions (Fig. 5). The phosphorylation of FtsH appears to be associated with Ca^2+^ response and/or FtsH complex formation as suggested by previous studies (Stael et al. 2012; Kato and Sakamoto 2019). In this study, we showed that the phosphorylation of thylakoid membrane proteins can be explored by combining the Phos-tag analysis with biochemical approaches, such as subfractionation. Future research using the Phos-tag analysis can uncover the involvement of protein phosphorylation in the function of proteins.

## Supplementary Information

Below is the link to the electronic supplementary material.

Supplementary file1 (PPTX 7243 kb)

## Data Availability

Not applicable.

## Abbreviations

GC: Grana core
GM: Grana margin
LHCI: Light-harvesting complex of photosystem I
LHCII: Light-harvesting complex of photosystem II
PSII: Photosystem II
PSI: Photosystem I
SL: Stroma lamellae
2D SDS-PAGE: Two-dimensional (2D) SDS-PAGE
GDN: Detergent glyco-diosgenin

## References

[CR1] Allahverdiyeva Y, Suorsa M, Tikkanen M, Aro EM (2015). Photoprotection of photosystems in fluctuating light intensities. J Exp Bot.

[CR2] Armbruster U, Labs M, Pribil M, Viola S, Xu W, Scharfenberg M, Hertle AP, Rojahn U, Jensen PE, Rappaport F, Joliot P, Dörmann P, Wanner G, Leister D (2013). *Arabidopsis* CURVATURE THYLAKOID1 proteins modify thylakoid architecture by inducing membrane curvature. Plant Cell.

[CR3] Baena-González E, Aro EM (2002). Biogenesis, assembly and turnover of photosystem II units. Philos Trans R Soc Lond B Biol Sci.

[CR4] Bellafiore S, Barneche F, Peltier G, Rochaix JD (2005). State transitions and light adaptation require chloroplast thylakoid protein kinase STN7. Nature.

[CR5] Bonardi V, Pesaresi P, Becker T, Schleiff E, Wagner R, Pfannschmidt T, Jahns P, Leister D (2005). Photosystem II core phosphorylation and photosynthetic acclimation require two different protein kinases. Nature.

[CR6] Crepin A, Caffarri S (2015). The specific localizations of phosphorylated Lhcb1 and Lhcb2 isoforms reveal the role of Lhcb2 in the formation of the PSI-LHCII supercomplex in *Arabidopsis* during state transitions. Biochim Biophys Acta.

[CR7] Damkjaer JT, Kereïche S, Johnson MP, Kovacs L, Kiss AZ, Boekema EJ, Ruban AV, Horton P, Jansson S (2009). The photosystem II light-harvesting protein Lhcb3 affects the macrostructure of photosystem II and the rate of state transitions in *Arabidopsis*. Plant Cell.

[CR8] Dumas L, Chazaux M, Peltier G, Johnson X, Alric J (2016). Cytochrome *b*_*6*_*f* function and localization, phosphorylation state of thylakoid membrane proteins and consequences on cyclic electron flow. Photosynth Res.

[CR9] Eng JK, McCormack AL, Yates JR (1994). An approach to correlate tandem mass spectral data of peptides with amino acid sequences in a protein database. J Am Soc Mass Spectrom.

[CR10] Fristedt R, Vener AV (2011). High light induced disassembly of photosystem II supercomplexes in *Arabidopsis* requires STN7-dependent phosphorylation of CP29. PLoS ONE.

[CR11] Fristedt R, Willig A, Granath P, Crèvecoeur M, Rochaix JD, Vener AV (2009). Phosphorylation of photosystem II controls functional macroscopic folding of photosynthetic membranes in *Arabidopsis*. Plant Cell.

[CR12] Grieco M, Suorsa M, Jajoo A, Tikkanen M, Aro EM (2015). Light-harvesting II antenna trimers connect energetically the entire photosynthetic machinery—including both photosystems II and I. Biochim Biophys Acta.

[CR13] Grieco M, Jain A, Ebersberger I, Teige M (2016). An evolutionary view on thylakoid protein phosphorylation uncovers novel phosphorylation hotspots with potential functional implications. J Exp Bot.

[CR14] Ihnatowicz A, Pesaresi P, Lohrig K, Wolters D, Müller B, Leister D (2008). Impaired photosystem I oxidation induces STN7-dependent phosphorylation of the light-harvesting complex I protein Lhca4 in *Arabidopsis thaliana*. Planta.

[CR15] Ingelsson B, Vener AV (2012). Phosphoproteomics of *Arabidopsis**chloroplasts* reveals involvement of the STN7 kinase in phosphorylation of nucleoid protein pTAC16. FEBS Lett.

[CR16] Kato Y, Sakamoto W (2009). Protein quality control in chloroplasts: a current model of D1 protein degradation in the photosystem II repair cycle. J Biochem.

[CR17] Kato Y, Sakamoto W (2014). Phosphorylation of photosystem II core proteins prevents undesirable cleavage of D1 and contributes to the fine-tuned repair of photosystem II. Plant J.

[CR18] Kato Y, Sakamoto W (2018). FtsH Protease in the thylakoid membrane: physiological functions and the regulation of protease activity. Front Plant Sci.

[CR19] Kato Y, Sakamoto W (2019). Phosphorylation of the chloroplastic metalloprotease FtsH in *Arabidopsis* characterized by Phos-Tag SDS-PAGE. Front Plant Sci.

[CR20] Kato Y, Sun X, Zhang L, Sakamoto W (2012). Cooperative D1 degradation in the photosystem II repair mediated by chloroplastic proteases in *Arabidopsis*. Plant Physiol.

[CR21] Kinoshita E, Takahashi M, Takeda H, Shiro M, Koike T (2004). Recognition of phosphate monoester dianion by an alkoxide-bridged dinuclear zinc(II) complex. Dalton Trans.

[CR22] Kinoshita E, Kinoshita-Kikuta E, Takiyama K, Koike T (2005). Phosphate-binding tag, a new tool to visualize phosphorylated proteins. Mol Cell Proteomics.

[CR23] Kinoshita E, Kinoshita-Kikuta E, Matsubara M, Yamada S, Nakamura H, Shiro Y, Aoki Y, Okita K, Koike T (2008). Separation of phosphoprotein isotypes having the same number of phosphate groups using phosphate-affinity SDS-PAGE. Proteomics.

[CR24] Longoni P, Douchi D, Cariti F, Fucile G, Goldschmidt-Clermont M (2015). Phosphorylation of the light-harvesting complex II isoform Lhcb2 is central to state transitions. Plant Physiol.

[CR25] Pesaresi P, Pribil M, Wunder T, Leister D (2011). Dynamics of reversible protein phosphorylation in thylakoids of flowering plants: the roles of STN7, STN8 and TAP38. Biochim Biophys Acta.

[CR26] Porra RJ, Thompson WA, Kriedemann PE (1989). Determination of accurate extinction coefficients and simultaneous equations for assaying chlorophylls a and b extracted with four different solvents: verification of the concentration of chlorophyll standards by atomic absorption spectroscopy. Biochim Biophys Acta.

[CR27] Pribil M, Pesaresi P, Hertle A, Barbato R, Leister D (2010). Role of plastid protein phosphatase TAP38 in LHCII dephosphorylation and thylakoid electron flow. PLoS Biol.

[CR28] Pribil M, Sandoval-Ibáñez O, Xu W, Sharma A, Labs M, Liu Q, Galgenmüller C, Schneider T, Wessels M, Matsubara S, Jansson S, Wanner G, Leister D (2018). Fine-tuning of photosynthesis requires CURVATURE THYLAKOID1-mediated thylakoid plasticity. Plant Physiol.

[CR29] Rantala M, Tikkanen M, Aro EM (2017). Proteomic characterization of hierarchical megacomplex formation in *Arabidopsis* thylakoid membrane. Plant J.

[CR30] Reiland S, Finazzi G, Endler A, Willig A, Baerenfaller K, Grossmann J, Gerrits B, Rutishauser D, Gruissem W, Rochaix JD, Baginsky S (2011). Comparative phosphoproteome profiling reveals a function of the STN8 kinase in fine-tuning of cyclic electron flow (CEF). Proc Natl Acad Sci USA.

[CR31] Sakamoto W, Zaltsman A, Adam Z, Takahash Y (2003). Coordinated regulation and complex formation of *yellow variegated1* and *yellow variegated2*, chloroplastic FtsH metalloproteases involved in the repair cycle of photosystem II in *Arabidopsis* thylakoid membranes. Plant Cell.

[CR32] Samol I, Shapiguzov A, Ingelsson B, Fucile G, Crèvecoeur M, Vener AV, Rochaix JD, Goldschmidt-Clermont M (2012). Identification of a photosystem II phosphatase involved in light acclimation in *Arabidopsis*. Plant Cell.

[CR33] Schönberg A, Rödiger A, Mehwald W, Galonska J, Christ G, Helm S, Thieme D, Majovsky P, Hoehenwarter W, Baginsky S (2017). Identification of STN7/STN8 kinase targets reveals connections between electron transport, metabolism and gene expression. Plant J.

[CR34] Shapiguzov A, Ingelsson B, Samol I, Andres C, Kessler F, Rochaix JD, Vener AV, Goldschmidt-Clermont M (2010). The PPH1 phosphatase is specifically involved in LHCII dephosphorylation and state transitions in *Arabidopsis*. Proc Natl Acad Sci USA.

[CR36] Stael S, Rocha AG, Wimberger T, Anrather D, Vothknecht UC, Teige M (2012). Cross-talk between calcium signalling and protein phosphorylation at the thylakoid. J Exp Bot.

[CR37] Tikkanen M, Aro EM (2012). Thylakoid protein phosphorylation in dynamic regulation of photosystem II in higher plants. Biochim Biophys Acta.

[CR38] Tikkanen M, Piippo M, Suorsa M, Sirpiö S, Mulo P, Vainonen J, Vener AV, Allahverdiyeva Y, Aro EM (2006). State transitions revisited-a buffering system for dynamic low light acclimation of *Arabidopsis*. Plant Mol Biol.

[CR39] Tikkanen M, Nurmi M, Kangasjärvi S, Aro EM (2008). Core protein phosphorylation facilitates the repair of photodamaged photosystem II at high light. Biochim Biophys Acta.

[CR40] Wang L, Kim C, Xu X, Piskurewicz U, Dogra V, Singh S, Mahler H, Apel K (2016). Singlet oxygen- and EXECUTER1-mediated signaling is initiated in grana margins and depends on the protease FtsH2. Proc Natl Acad Sci USA.

[CR41] Yu F, Park S, Rodermel SR (2004). The *Arabidopsis* FtsH metalloprotease gene family: interchangeability of subunits in chloroplast oligomeric complexes. Plant J.

[CR42] Zaltsman A, Ori N, Adam Z (2005). Two types of FtsH protease subunits are required for chloroplast biogenesis and photosystem II repair in *Arabidopsis*. Plant Cell.

