# Supplementary material for: Phos-tag-based approach to study protein phosphorylation in the thylakoid membrane
Source: Photosynth Res. 2020 Dec 2;147(1):107–24. doi: 10.1007/s11120-020-00803-1 (PMC7728655; doi:10.1007/s11120-020-00803-1)
Supplement: Supplementary file 1 — Supplementary file1 (PPTX 7243 kb) [file 11120_2020_803_MOESM1_ESM.pptx]

## Slide 1
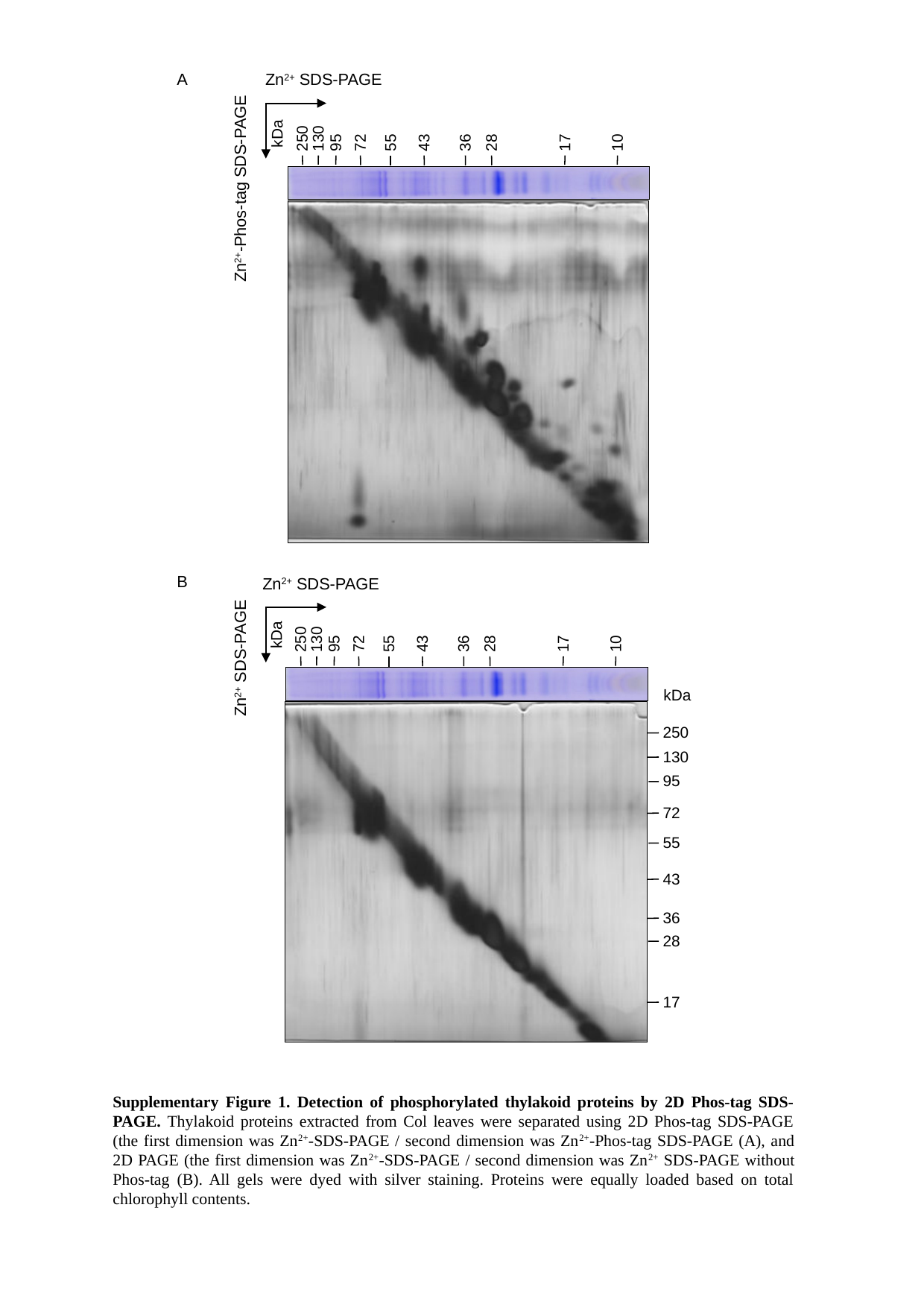

A
Zn2+ SDS-PAGE
250
130
95
72
55
36
28
17
10
43
kDa
Zn2+-Phos-tag SDS-PAGE
B
Zn2+ SDS-PAGE
250
130
95
72
55
36
28
17
10
43
kDa
Zn2+ SDS-PAGE
kDa
250
130
95
72
55
43
36
28
17
Supplementary Figure 1. Detection of phosphorylated thylakoid proteins by 2D Phos-tag SDS-PAGE. Thylakoid proteins extracted from Col leaves were separated using 2D Phos-tag SDS-PAGE (the first dimension was Zn2+-SDS-PAGE / second dimension was Zn2+-Phos-tag SDS-PAGE (A), and 2D PAGE (the first dimension was Zn2+-SDS-PAGE / second dimension was Zn2+ SDS-PAGE without Phos-tag (B). All gels were dyed with silver staining. Proteins were equally loaded based on total chlorophyll contents.

## Slide 2
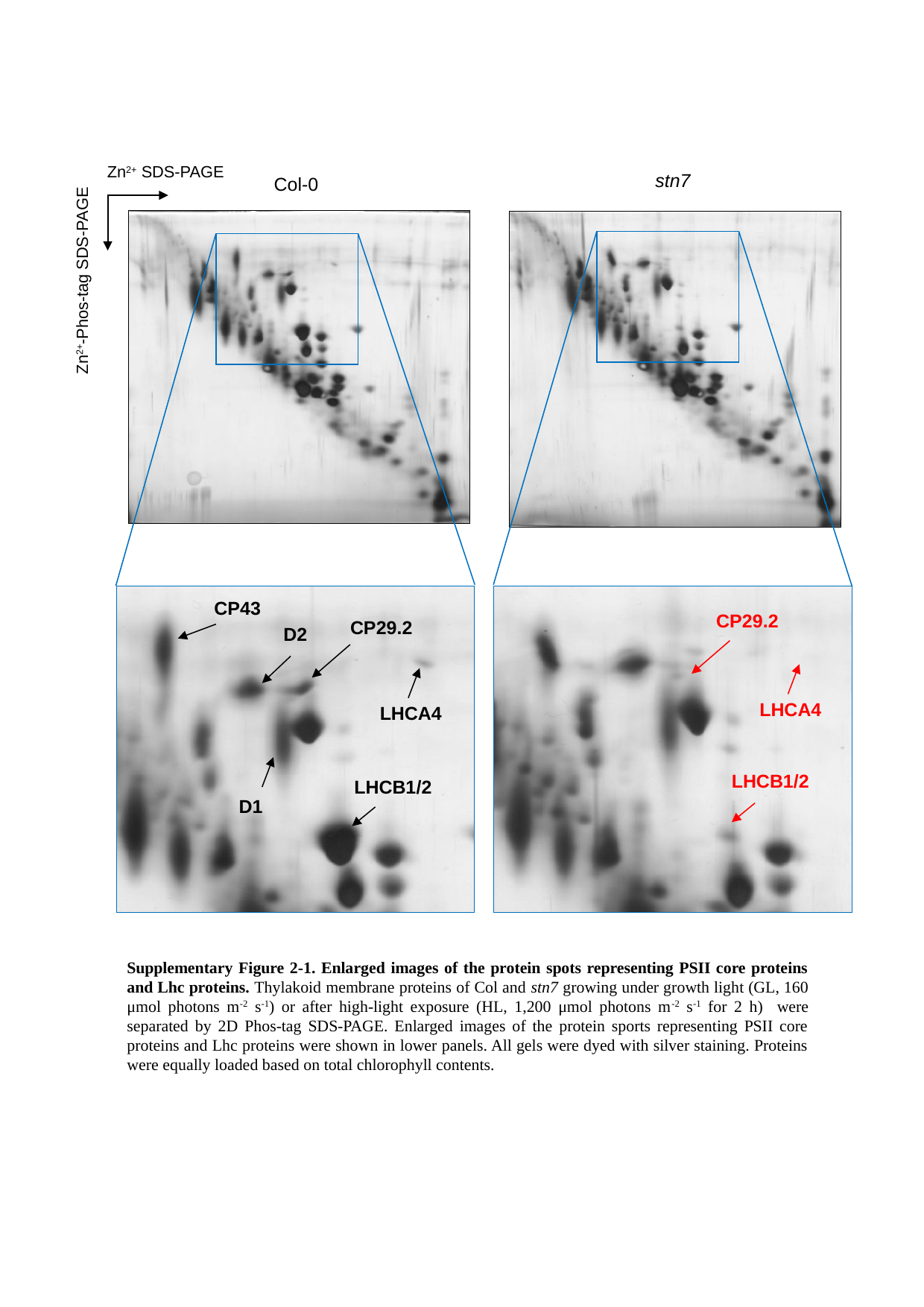

Zn2+ SDS-PAGE
stn7
Col-0
Zn2+-Phos-tag SDS-PAGE
CP43
CP29.2
CP29.2
D2
LHCA4
LHCA4
LHCB1/2
LHCB1/2
D1
Supplementary Figure 2-1. Enlarged images of the protein spots representing PSII core proteins and Lhc proteins. Thylakoid membrane proteins of Col and stn7 growing under growth light (GL, 160 μmol photons m-2 s-1) or after high-light exposure (HL, 1,200 μmol photons m-2 s-1 for 2 h) were separated by 2D Phos-tag SDS-PAGE. Enlarged images of the protein sports representing PSII core proteins and Lhc proteins were shown in lower panels. All gels were dyed with silver staining. Proteins were equally loaded based on total chlorophyll contents.

## Slide 3
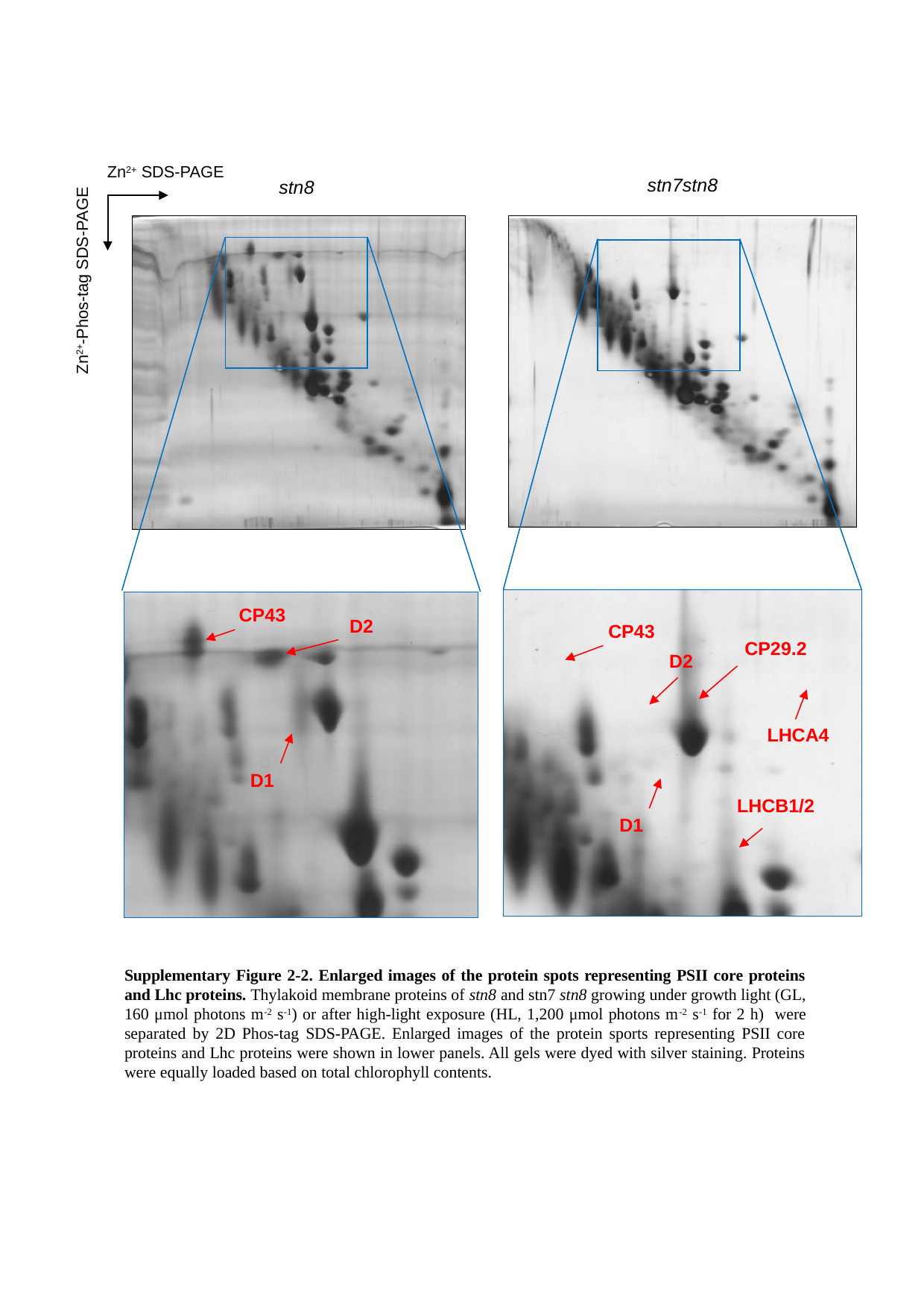

Zn2+ SDS-PAGE
Zn2+-Phos-tag SDS-PAGE
stn7stn8
stn8
CP43
D2
CP43
CP29.2
D2
LHCA4
D1
LHCB1/2
D1
Supplementary Figure 2-2. Enlarged images of the protein spots representing PSII core proteins and Lhc proteins. Thylakoid membrane proteins of stn8 and stn7 stn8 growing under growth light (GL, 160 μmol photons m-2 s-1) or after high-light exposure (HL, 1,200 μmol photons m-2 s-1 for 2 h) were separated by 2D Phos-tag SDS-PAGE. Enlarged images of the protein sports representing PSII core proteins and Lhc proteins were shown in lower panels. All gels were dyed with silver staining. Proteins were equally loaded based on total chlorophyll contents.

## Slide 4
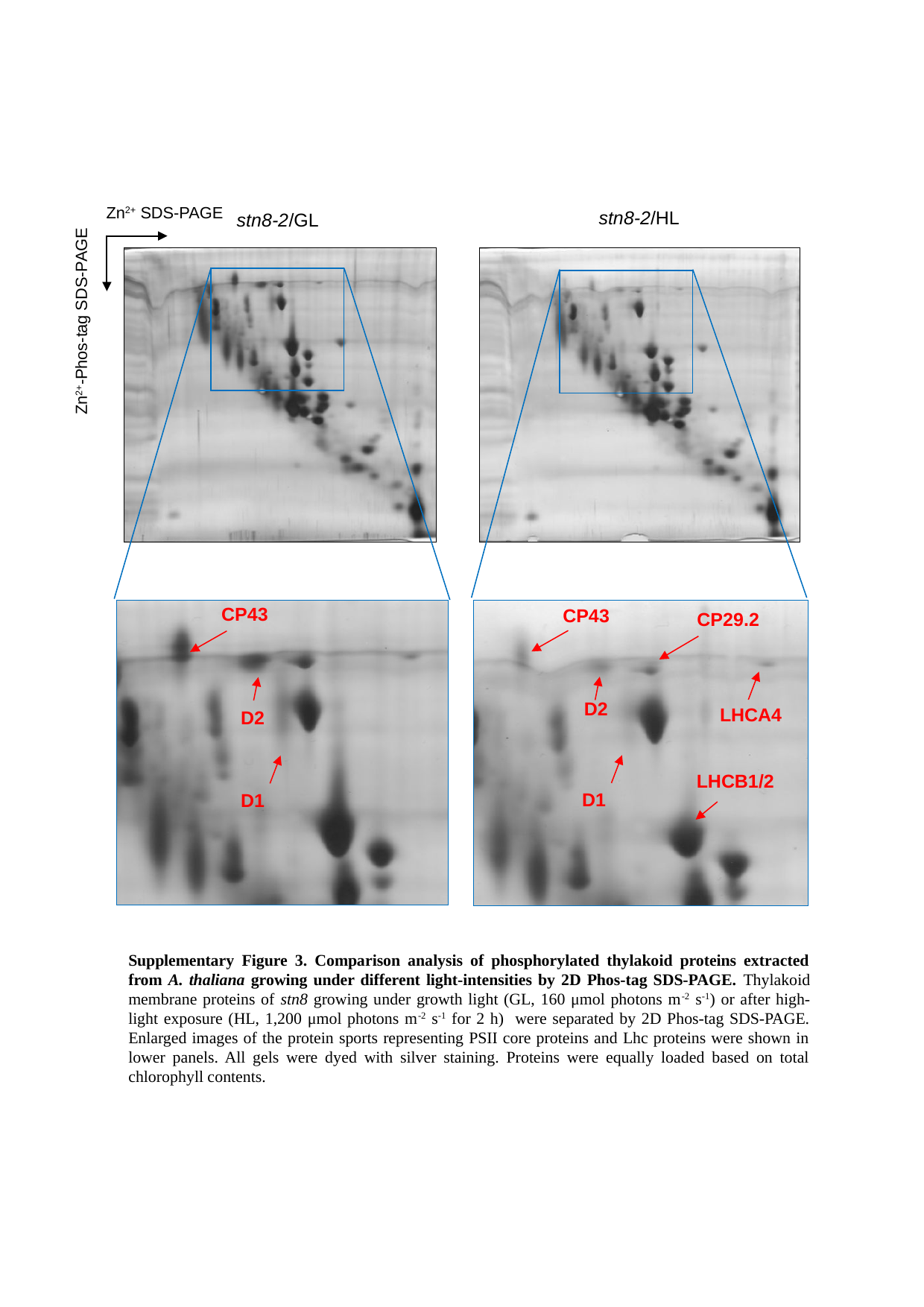

Zn2+ SDS-PAGE
stn8-2/HL
stn8-2/GL
Zn2+-Phos-tag SDS-PAGE
CP43
CP43
CP29.2
D2
LHCA4
D2
LHCB1/2
D1
D1
Supplementary Figure 3. Comparison analysis of phosphorylated thylakoid proteins extracted from A. thaliana growing under different light-intensities by 2D Phos-tag SDS-PAGE. Thylakoid membrane proteins of stn8 growing under growth light (GL, 160 μmol photons m-2 s-1) or after high-light exposure (HL, 1,200 μmol photons m-2 s-1 for 2 h) were separated by 2D Phos-tag SDS-PAGE. Enlarged images of the protein sports representing PSII core proteins and Lhc proteins were shown in lower panels. All gels were dyed with silver staining. Proteins were equally loaded based on total chlorophyll contents.
